# Integrating voxel mapping with deep network-based point-line feature fusion for robust SLAM

**DOI:** 10.1371/journal.pone.0337917

**Published:** 2026-01-02

**Authors:** Yu Xin Qin, Wei Jie Zhou, Jun Liang Liu, Yu Chen, Xian Guang Wang, Liang Long Chen

**Affiliations:** 1 School of Electronics and Information, Zhengzhou University of Aeronautics, Zhengzhou, Henan, China; 2 Henan Key Laboratory of General Aviation Technology, Zhengzhou University of Aeronautics, Zhengzhou, Henan, China; 3 Collaborative Innovation Center of Aeronautics and Astronautics Electronic Information Technology, Zhengzhou, Henan, China; University of Florida, UNITED STATES OF AMERICA

## Abstract

The present study addresses the issues of feature loss and map consistency in visual SLAM under low-texture, low-light, and unstructured scenes by proposing an improved system based on ORB-SLAM3. The following innovative features are worthy of note: Firstly, a combination of custom voxel mapping and sparse SLAM is proposed for the purpose of enhancing matching robustness and 3D reconstruction quality in low-texture regions. Secondly, the utilization of a depth map neural network for the fusion of point and line features is suggested. Tests on public datasets and other unstructured scenes demonstrate that this method significantly improves joint feature matching efficiency, validating the complementary advantages of point and line features. The results indicate a considerable boost in both localization accuracy and environmental adaptability in challenging scenarios, setting the foundation for more reliable SLAM applications in real-world conditions.

## 1. Introduction

Visual Simultaneous Localization and Mapping (V-SLAM) technology is a fundamental component of environmental perception for autonomous mobile robots, and its development has significantly influenced innovation in fields such as autonomous driving, augmented reality, and intelligent service robots [1]. It is evident that classic visual SLAM systems are capable of achieving pose estimation and scene reconstruction through the processes of feature point extraction and geometric constraint optimization. However, due to the inherent limitations of traditional feature designs, they often experience feature matching degradation in scenarios with weak texture, drastic changes in lighting, and dynamic interference. This can lead to positioning drift or even system failure, resulting in crashes or map coverage re-registration [2]. The utilization of feature labelling methodologies founded upon deep learning has engendered a paradigm shift in the realm of visual SLAM. In contrast to manual feature extractors, which rely on low-order information such as image gradients and corners, learning-based feature detection networks implicitly model high-order semantic associations through end-to-end training. This approach has been shown to significantly improve the cross-view invariance and interference resistance of feature descriptors [3].

Ignatius et al. [4] proposed a dynamic feature selection mechanism based on motion consistency, which utilizes multi-hypothesis RANSAC to eliminate transient features in low-texture environments. This approach extended the tracking duration to 2.3 times that of traditional methods in desert scenes. Ma et al. [5] introduced a joint reprojection error function by coupling LBD line (Line Band Descriptor) segment descriptors with ORB point features, reducing pose estimation errors by 37% in indoor white-wall scenes. Chen et al. [6] employed a multi-scale Harris-FAST hybrid detection strategy, combined with pyramid optical flow, to enable cross-scale tracking, enhancing the continuity of matching in nighttime road scenes by 40%. Feng et al. [7] integrated Canny edge geometric constraints into the ORB descriptor space, achieving sub-pixel accuracy in feature matching on industrial, textureless metal surfaces. Bo et al. [8] utilized a lightweight graph convolutional network to model the topological relationships of feature points, aiding RANSAC in eliminating mismatches in vegetation scenes, resulting in a 21% improvement in F1-score. Mohadikar et al. [9] drove dynamic sampling using visual saliency, maintaining equivalent accuracy in scenes with fewer features, such as Gobi landscapes.

To address the aforementioned issues, this paper proposes a visual SLAM system that integrates depth feature geometric constraints. Our system is built upon the ORB3-SLAM framework, incorporating a custom voxel mapping module with a parallel frontend to enhance robustness in low-texture regions. Additionally, a depth map neural network is employed in the backend to fuse point and line features, thereby improving feature matching efficiency and realizing the complementary advantages of point and line features.

## 2. System overview

This chapter presents our specific SLAM system, which is primarily divided into two main aspects at a high level: the frontend parallel voxel mapping strategy and the backend GNN-based (Graph Neural Network) point-line fusion strategy.

### 2.1 Voxel mapping and sparse SLAM strategy

This research built a sparse subsystem based on ORB3-SLAM3 [10]. Each new keyframe generated by the SLAM module is used as input for the voxel mapping module. As a first step, the module converts the depth map $D_{i}$ of each new RGB-D keyframe into a high-precision 3D point cloud ${𝒫}_{i}$ in the camera coordinate system. If any of the following conditions are met, the pixel is ignored: δ≤0 or δ=NaN; δ exceeds the valid ranging range. Bilateral filtering [16,17] is performed on the entire depth map to smooth Gaussian noise while preserving edge structures. The filter kernel function [18,19] is:


$$D_{i}^{\prime }(u)=\frac{\text{1}}{W}\sum_{u_{j}\in 𝒩(u)}\exp\left(-\frac{||u-u_{j}|{|}^{2}}{2\sigma_{s}^{2}}-\frac{{\left({𝒟}_{i}(u)-{𝒟}_{i}(u_{j})\right)}^{2}}{2\sigma_{r}^{2}}\right){𝒟}_{i}(u_{j})$$ (1)

Among them, 𝒩(u) is the neighborhood of u, whose neighborhood can be adjusted through an adaptive window, $\sigma_{s}$ controls the spatial distance weight, $\sigma_{r}$ controls the depth difference weight, and W is the normalization factor. Next, the internal parameters of the camera need to be set. Assuming that the internal parameter matrix of each frame is:


$$𝒦=\left[\begin{array}{ccc} {}*20cf_{x} & 0 & c_{x}\\ 0 & f_{y} & c_{y}\\ 0 & 0 & 1 \end{array}\right]$$ (2)

Which $f_{x},f_{y}$ represent the horizontal and vertical focal lengths (in pixels); $c_{x},c_{y}$ represent the coordinates of the main point (center point) of the image, respectively. For each valid depth pixel $u=[u,v{]}^{T}$, its three-dimensional position $X_{c}\in {\mathbb{R}}^{3}$ in the camera coordinate system can be calculated as follows:


$$X_{c}=\left[\begin{array}{c} {}*20c(x-c_{x})\cdot \delta /f_{x}\\ (y-c_{y})\cdot \delta /f_{y}\\ \delta \end{array}\right]$$ (3)

Based on the basic assumption that a set of points within a small range approximately lie on a single plane, 3D plane fitting is performed on their neighborhoods in the depth map. On the depth map $D_{i}$, median difference is performed on each pixel to calculate its horizontal and vertical gradients. For each back-projected point $X_{c}$, select its corresponding 4-neighbourhood in the depth map: (u±1,v),(u,v±1). Back-project the corresponding 4 points to $\{X_{c}^{\left(j\right)}{\}}_{j=1}^{4}$, and then use the least squares fitting process to make the centroid of the neighborhood point set: $\overset{―}{X}=\frac{\text{1}}{N}\sum_{j=1}^{N}X_{j}$. Subtract the center of gravity from all points, and calculate the covariance of the resulting zero-mean coordinate matrix: $C=\frac{\text{1}}{N}Q^{T}Q\in {\mathbb{R}}^{3\times 3}$, and Q represents the zero-mean coordinate matrix obtained after subtracting the center of gravity from all points. By performing eigenvalue decomposition on C, the corresponding plane normal vectors can be obtained. Next, the areas are weighted and merged, and the four neighborhood normal vectors are weighted and averaged according to the triangle areas, where $A_{j}$ is the corresponding triangle area:


$$n_{u}=\frac{\sum_{j=1}^{4}A_{j}n^{\left(j\right)}}{\left||\sum_{j=1}^{4}A_{j}n^{\left(j\right)}|\right|}$$ (4)

Select voxel resolution r and divide the three-dimensional space into an equilateral cube grid. For the custom Octree [20], find the voxel node to which each point $X_{w}$ belongs, accumulate its coordinate, color, and normal vector statistics, and simultaneously update the node’s average value $\overset{―}{X}$, average normal vector $\overset{―}{n}$, and count N. In accordance with the prevailing camera position, rays are emitted to each point, and the voxels along the ray path are marked as ‘empty’; the voxel where the point is located is updated to ‘occupied’, and the occupancy probability is updated using the Bayesian filtering formula. At this point, calculate the distance Φ between each voxel and the point cloud surface, truncate it within the range [−μ,+μ], and simultaneously update the TSDF (Truncated Signed Distance Function) value and synchronize the weight:


$$\Phi_{new}=\frac{w_{old}\Phi_{old}+w_{meas}\phi }{w_{old}+w_{meas}}$$ (5)

In order to maintain consistency between the sparse SLAM map and the voxel map, the voxel map is reconstructed or adjusted when the SLAM map or its base pose map undergoes a global adjustment (e.g., when a loop is detected), as illustrated in S1 Fig. In the event of the SLAM front end detecting a closed loop or performing a global BA (Bundle Adjustment), the keyframe pose undergoes a change.

As illustrated in S1 Fig. The point cloud processing results derived from the TUM RGB-D dataset are presented. Panel (a) corresponds to the original point cloud map, while Panel (b) represents the plane extraction result map. The original point cloud is generated from color images and depth maps, preserving the geometric structure and texture information of the scene relatively intact. Typical structural surfaces, including the ground, walls, and tabletops, are clearly demonstrated, while locally sparse areas, caused by occlusion or missing depth data, are also revealed. The front-end method proposed in this paper performs multi-scale RANSAC plane detection, normal estimation, and point cloud filtering on the original point cloud. Each detected plane is marked with a different color, and a normal vector arrow is added at its center to indicate its spatial orientation. The experiment extracted multiple significant voxel planes, which covered most of the main geometric regions in the scene. Points that have not been allocated to any plane are displayed in grey, with a primary concentration observed in the vicinity of boundaries, regions exhibiting weak textures, or areas where discontinuous depth information is present.

### 2.2 Depth map neural network point-line fusion strategy

Inspired by the idea that contextual relationships between features can improve matching performance, the single-point feature representation used in the original system is unable to accurately express the three-dimensional structural features of a scene. In order to address this issue, the present study employs traditional feature detectors to extract point and line features and their descriptors, subsequently encoding them into a unified wireframe structure. The key points, dense descriptors and lines from two images have been extracted and unified into two wireframe structures. In GNN [21–23], the features of the nodes in the two wireframe structures are enriched through self, line, and cross attention, as illustrated in S2 Fig.

The objective of the GNN module is to amalgamate the visual and spatial information of each feature and enable interaction between all features, irrespective of their original receptive fields. The resulting output comprises a set of updated descriptors, which are enriched by knowledge edges between related features within and between images and within nodes connected by lines.

It is proposed that images A and B be regarded as a pair of images. For each image, the network’s input is constituted by a set of nodes p with coordinates $(x_{p},y_{p})$, confidence scores $s_{p}$, visual descriptors $d^{vis}\in {\mathbb{R}}^{D}$, and a set of line segments ℒ. Each key point and line segment endpoint is converted into a node 𝒱 in the graph, thus forming graph G=(𝒱,ℰ). The connection relationships (line segments) between nodes form the edge set ℰ. For each node $u_{i}\in 𝒱$, its initial representation is a fusion of position and visual information:


$${\tilde{f}}_{i}^{\left(0\right)}=\phi_{v}(d_{i})+\phi_{s}(p_{i})$$ (6)

Where $\phi_{v}$ represents visual feature extraction, $\phi_{s}$ represents position encoding MLP, and $d_{i}$ represents descriptors. In the online feature information transmission end, through the connecting lines in the online frame structure, this study allow the $i^{th}$ node to utilize the local edge connections with the set of adjacent nodes $p_{i}$ and search for the same type of connection in another image. The implementation of this mechanism is effected by means of an update to the $m^{th}$ line feature information transmission end. The update is responsible for the encoding of the information contained within the two endpoint features, $x_{i}$ and $x_{j}$, and the corresponding endpoint positions. The purpose of this process is to strengthen spatial structure perception:


$${\tilde{f}}_{i}^{\left(t+1\right)}={\tilde{f}}_{i}^{\left(t\right)}+\frac{\text{1}}{\left|{ℰ}_{i}\right|}\sum_{j\in {ℰ}_{i}}\Phi_{t}\left(\left[{\tilde{f}}_{i}^{\left(t\right)}||{\tilde{f}}_{j}^{\left(t\right)}||e_{ij}\right]\right)$$ (7)

Where ${\tilde{f}}_{i}^{\left(t\right)}\in {\mathbb{R}}^{d}$ denotes the feature vector of node i at layer t, with dimension d (in experiments d=128). ${ℰ}_{i}$ represents the set of adjacent nodes connected to node i, and $\frac{\text{1}}{\left|{ℰ}_{i}\right|}$ denotes the neighborhood normalization factor, which ensures that the update magnitudes remain consistent even when different nodes have different numbers of adjacent nodes. $e_{ij}$ denotes the spatial encoding of an edge, representing the edge feature between node i and j, which contains spatial and geometric information (such as relative position, direction vector, and distance), and $d_{e}$ is typically set to 32 or 64. $\Phi_{t}$ denotes the edge feature updater, which is a one-layer or two-layer MLP that maps and unifies dimensional information, enabling local feature aggregation. The output dimension of each layer t is consistent with its input dimension. Finally, the system matches point and line features through dual Softmax.

### 2.3 Cross-map adhesion strategy

Subsequent to the completion of the local fusion update of point and line features by the depth map neural network, in order to address the issue of global consistency under cross-view or occlusion conditions, a cross-map adhesion strategy is innovatively introduced. The core mechanism of this module is to construct a bidirectional recurrent comparison architecture between two graphs, thereby achieving deep interaction of cross-view information through an iterative structural alignment process. The proposed methodology employs a dynamic attention mechanism to identify potential associations between disparate views. Subsequently, a recurrent consistency loss function is utilized to impose constraints on the topological correspondence of graph structures, thereby establishing a stable feature matching mapping.

Assume that after GNN update, the point features are $F_{p}^{\left(A\right)}\in {\mathbb{R}}^{N_{p}\times d}$ and $F_{p}^{\left(B\right)}\in {\mathbb{R}}^{M_{p}\times d}$, and the line features are $F_{l}^{\left(A\right)}\in {\mathbb{R}}^{N_{l}\times d}$ and $F_{l}^{\left(B\right)}\in {\mathbb{R}}^{M_{l}\times d}$, where d is the feature dimension and $N_{p},M_{p},N_{l},M_{l}$ is the number of points and lines in the two graphs. This research jointly denote them as: $F^{\left(A\right)}=[F_{p}^{\left(A\right)};F_{l}^{\left(A\right)}]$, $F^{\left(B\right)}=[F_{p}^{\left(B\right)};F_{l}^{\left(B\right)}]$, which denote the node feature matrices of the source graph and the target graph, respectively. Then construct an initial similarity matrix through a learnable attention comparison function:


$$S^{\left(0\right)}=Softmax\left(\frac{F^{\left(A\right)}\cdot {\left(F^{\left(B\right)}\right)}^{T}}{\sqrt{d}}\right)$$ (8)

For Formula (8), the elements of the dot product result are similarity scores between nodes, and $\sqrt{d}$ represents a scaling factor, which is used to avoid excessively large inner product values in high dimensions. In this study, d is typically set to 128, so the scaling factor is approximately 11.31. Softmax(·) is normalized along the row dimension, i.e., the sum of elements in each row is 1:


$$\sum_{j=1}^{N_{B}}S_{ij}^{\left(0\right)}=1,\;S_{ij}^{\left(0\right)}\in [0,1]$$ (9)

Where $S^{\left(0\right)}$ denotes the initial cross-graph similarity matrix, and its values can be regarded as the matching probability from node i∈A to node j∈B. Following the implementation of the Softmax(·) row normalization operation, the probability of each source image node matching the target image can be simulated. Consequently, a cyclic adhesion update mechanism is introduced. In round t, the following updates are performed by this module for each feature:


$$\{{}_{F_{t+1}^{\left(B\right)}=LayerNorm(F_{t}^{\left(B\right)}+MLP({\left(S^{\left(t\right)}\right)}^{T}\cdot F_{t}^{\left(A\right)}))}^{F_{t+1}^{\left(A\right)}=LayerNorm(F_{t}^{\left(A\right)}+MLP(S^{\left(t\right)}\cdot F_{t}^{\left(B\right)}))}$$ (10)

Where $S^{\left(t\right)}\in {\mathbb{R}}^{N_{A}\times N_{B}}$ represents the $t^{th}$ round cross-graph matching weight matrix, with the sum of elements in each row being 1. $S^{\left(t\right)}\cdot F_{t}^{\left(B\right)}$ denotes the cross-graph weighted aggregation operation, i.e., each node in A aggregates feature from B. MLP(·) represents a two-layer perceptron model for feature fusion, and LayerNorm(·) maintains feature stability and numerical balance. At this point, $S^{\left(t\right)}$ acts as a weight bridge matrix to ‘glue’ the two graphs together, while retaining the residual information from the previous round to form the gluing path. After T rounds of updates, the final stable glued feature $F_{T}^{\left(A\right)},F_{T}^{\left(B\right)}$ are output. Which is used to construct the final matching matrix:


$$S^{*}=Softmax\left(\frac{F_{T}^{\left(A\right)}\cdot {\left(F_{T}^{\left(B\right)}\right)}^{T}}{\sqrt{d}}\right)$$ (11)

The result is then Sinkhorn normalized [24] and used to decode the final point-line matching pairs, as shown in Algorithm 1.

Algorithm 1. Overall Voxel-GNN SLAM System Pipeline

**1. Initialization**: Load camera intrinsics K,depth threshold $\tau_{K}$,iteration count $T_{1}$,initialize voxel map $M_{voxel}$,and trajectory T.

**2. For** each RGB-D keyframe $I_{K}=\{{I^{RGB}}_{K},D_{K}\}$,perform the following steps:

 a) **Back-project** depth $D_{K}$ into 3D space via camera intrinsics (see Eq. (3)) to obtain point cloud $P_{K}$.

 b) **Denoise** point cloud using bilateral filter.

 c) **Estimate** local surface normal via plane fitting (see Eq. (4)), and update voxel occupancy using TSDF and Bayesian filtering (see Eq. (5)).

 d) **If** loop closure or pose adjustment is triggered, **re-align** voxel map accordingly.

**3. For** each image pair $\left(I_{i,}I_{j}\right)$ in candidate set:

 a) **Extract** ORB points p, lines I,descriptors d,and build graph G for both images.

 b) **Pass** graph G through GNN (Eq. (6),(7)) to obtain updated features $h_{i},h_{j}$.

 c) **Compute** attention matrix $S=Attention\left(h_{i},h_{j}\right)$, apply row-wise Softmax(·) normalization (Eq. (8)).

 d) **Repeat** for t = 1 to T:

 e) **Update** features:

 $h_{i}\leftarrow h_{i}+MLP(S\times h_{j})$

 $h_{j}\leftarrow h_{j}+MLP(S^{T}\times h_{i})$

 (see Eq. (10))

**4. Match** final features via Sinkhorn normalization (Eq. (11)) to get point-line correspondences M.

**5. Return** optimized camera trajectory T, dense voxel map $M_{voxel}$, and matching result M.

The adhesion strategy under scrutiny in this study is predicated on the construction of structural flow paths between graphs, thereby facilitating reasonable matching even in circumstances where certain nodes exhibit weak local features, through the leveraging of other structures by means of network conduction. The overall structure is complementary to the point-line multi-layer fusion strategy referenced earlier in this paper. The former achieves strong structural representation within the spatial domain, while the latter reinforces information through cross-graph cycling, demonstrating excellent robustness under extreme conditions such as lighting changes, perspective differences, and texture degradation.

### 2.4 Real trajectory generation strategy

One challenging task in line matching is to generate high-quality labels that handle line fragmentation, allocation, and partial visibility. To obtain true trajectory point matches, our research uses camera pose and depth to reproject keypoints from one image to another, and adds new matches whenever the reprojection falls within the small neighborhood of an existing keypoint.

For the association and matching of line features, depth is also utilized, but the settings are more complex. Suppose images A and B contain M and N line segments, respectively, and their indices are marked as A:{1,...M} and B:{1,...N}, which represent the generated real path trajectory line matching as $M^{l}=\{(i,j\}\subset A\times B$. For each segment S detected in image A, sample K points along the neighborhood within the line segment: $\left[x_{i,1}^{A},\ldots ,x_{i,K}^{A}\right]$. If point i has no depth, or if its projection $x_{i}^{B}$ in another image has no depth, it is deemed an invalid point. Similarly, if point i is partially obscured, resulting in unclear information, it is also deemed an invalid point. Finally, by comparing the depth $d(X_{i})$ of the 3D projection point $X_{i}$ of point $x_{i}^{A}$ with its expected depth $d^{B}$ in image B, the line feature matching is detected once:


$$Occluded=\left|\frac{d(X_{i})-d^{B}}{d^{B}}\right|>\tau$$ (12)

Where τ defines the tolerance threshold for depth changes. Segments with more than 50% invalid points are marked as IGNORE points.

Next, system generates a proximity matrix: $C^{B}\in {\mathbb{N}}^{M\times N}$, tracking how many sampling points of line i in image A are reprojected to the vicinity of line j in B:


$$C_{i,j}^{B}=\sum_{k=1}^{K}r(valid(x_{i,k}^{B})\wedge d_{⊥}(x_{i,k}^{B},l_{j}^{B})<T_{dist})$$ (13)

In the context of the image under consideration, the symbol r(⬝) denotes the indicator function, the symbol $d_{⊥}(⬝)$ denotes the vertical distance from the given point to the line, and the symbol $T_{dist}$ is used to denote the pixel distance threshold that controls the strictness of the true trajectory. Finally, the Hungarian algorithm [11] is used to solve the assignment problem defined by the proximity similarity matrix. The resulting assignment pair $(i,j)\in M^{l}$ is the matched feature, while all unassigned valid entries I⊆A and J⊆B are marked as unmatched.

### 2.5 Closed-loop optimization based on word bags

In order to reduce the cumulative drift caused by the visual odometer, loop detection matches the current submap’s historical keyframes with the keyframes obtained from the tracking thread using a visual bag-of-words (BoW) model. The system then calculates the similarity score between the BoW vector of the current keyframe and the BoW vector of the historical keyframes to determine loops. This process optimizes the map and camera motion trajectories. The loop detection module is also divided into two parts: position identification and geometric verification.

Map points are three-dimensional feature points that are reconstructed from images. This reconstruction is achieved using methods such as triangulation, and the resulting points contain their spatial positions and corresponding observation key frame information. In the event of a map point being observed by multiple key frames simultaneously, a co-visibility relationship is established between the observation frames. Keyframes are modelled as nodes, and the co-visibility relationships between keyframes are modelled as edges, thereby constructing a co-visibility graph. Consequently, edges with higher co-visibility relationship strengths are subjected to further filtration to form an essential graph, thereby enhancing the stability and robustness of the graph structure.

To achieve effective matching between images, this study employs the BoW model based on ORB features. The current frame is compared with key frames in the BoW database using similarity matching, and the key frame with the highest similarity is selected as the candidate matching frame. Specifically, the BoW vector v of the current frame is first extracted and its similarity is calculated with the BoW vectors w of each key frame in the common view. The calculation formula is as follows:


$$s(w-v)=2\sum_{i=1}^{N}|w_{i}|+|v_{i}|-|w_{i}-v_{i}|$$ (14)

For each key frame in the co-view, the minimum matching score $S_{min}$ is obtained through similarity comparison and used as the reference threshold for loop detection. The fundamental principle of loop detection hinges upon ascertaining the existence of duplicate regions between the present frame and historical imagery. The subsequent identification of these duplicate observation features is pivotal in determining loop detection. In order to circumvent any potential interference, it is imperative to initially exclude all key frames contained within the current frame’s co-view. Subsequently, an inverted index is created for the terms contained in the current frame’s BoW vector, with the objective of obtaining the associated historical key frames. The number of term bags shared between each key frame and the current frame is then counted. Select the value M with the highest number of similar word bags through sorting, and form a candidate set $KF_{1}$ consisting of key frames with a number of similar word bags not less than 0.8×M. Next, calculate the BoW matching score between the key frames in $KF_{1}$ and the current frame, and retain the key frames with matching scores higher than $S_{min}$ to form a new set $KF_{2}$. Furthermore, the total BoW matching score $S_{total}$ between the common key frames of each candidate frame in $KF_{2}$ and the current frame is calculated. Only key frames with a score greater than $0.75\times S_{total}$ are retained to form the final candidate set $KF_{3}$. If a loop occurs more than three times in a row during detection, the candidate set is further reduced to finally determine the set of candidate key frames $K_{final}$ for the loop, which significantly improves the accuracy and robustness of loop detection.

Subsequent to the acquisition of the candidate frame set $K_{final}$, geometric consistency verification is imperative in order to ascertain the authenticity of its loop-like configuration. The RANSAC method is utilized to estimate geometric constraints for matching feature points between the current frame and the reference frame. A nonlinear optimization model is constructed to solve for the rotation matrix, relative translation vector, and scale factor between the two frames. Assume that the pose transformation between the current frame and the loopback candidate frame is $T_{rc}$, the 3D point in the current frame coordinate system is $P_{c}$, its corresponding pixel coordinate in the current frame is $p_{c}$, the 3D coordinate of the matched point in the loopback frame is $P_{r}$, and its corresponding pixel coordinate is $p_{r}$. Then, the reprojection error is defined as:


$$e_{rc}=p_{r}-KT_{rc}P_{c}$$ (15)

$$e_{cr}=p_{c}-KT_{cr}P_{r}$$ (16)

In the circumstance that the reprojection error of all matching points is found to be less than the preset threshold, the system will determine that the point pair is an inlier. Once the number of inliers reaches the set standard, the relative pose transformation between the current frame and the reference frame can be estimated, and further nonlinear optimization can be performed. It is evident that, through continuous iteration of matching points and optimization, if the final number of inliers meets the robustness requirements of loop detection, it can be determined that the loop has been successfully detected.

## 3. Results & Discussion

### 3.1 Experimental setup

All experiments were conducted on a laptop equipped with a 13th Gen Intel® Core™ i7-13700H processor and an NVIDIA GeForce RTX 4060 GPU. The robotics experiment platform uses the MiniuGv 10C chassis vehicle, running in an Ubuntu 20.04 virtual system based on the ARM architecture. The visual information acquisition module employs the Orbbec Astra Pro Plus depth camera, enabling real-time acquisition of high-precision RGB-D data. The experimental setup is shown in S3 Fig. Experimental Setup Diagram.

This study pre-trained the model on publicly available datasets, gradually increasing the monotonicity difficulty and accelerating convergence. For image pair selection, this study used image pairs with at least 10% 3D point overlap and adjusted each image to 640 × 640 pixels. The wireframe threshold d for merging nodes was set to 3 pixels, When the system generates real trajectory paths, the τ depth change tolerance threshold is set to 0.1 to determine the degree of occlusion of line sampling points in another image; $T_{dist}$ = 5 is used to represent the vertical distance threshold between a point and a line, which determines whether the reprojected point of the line sampling point in another image is close to the target line segment. When the vertical distance between the reprojected point and the target line segment is less than this threshold, the point is determined to be “close” to the target line segment and is included in the image proximity matrix $C^{B}$ or $C^{A}$. For the minimum overlap threshold $\tau_{overl}$ of line features, the minimum overlap threshold is set to 0.2 to assess the validity of line matching.

In the training of the depth GNN module, the matching threshold is set to η=2, the feature size within the network is D=256, and the learning rate of the single-response pre-training in the module is $10^{-4}$. To limit the computational and time costs during training, the maximum number of line segments per image is set to 250.

### 3.2 Point-line strategy matching evaluation

The initial investigation comprised a systematic comparative experiment to ascertain the relevance of point-line feature matching. In light of prevalent, intricate scenarios such as occlusion, background modifications, and partial visibility, the process of stable matching of line segments in three-dimensional space remains to be significantly challenging. The present study therefore undertakes a comprehensive analysis of the matching performance between points and lines. As illustrated in S4 Fig. The outcomes of the matching experiment employing this back-end point-line strategy vary across different test scenarios.

S4 Fig. shows the experimental results indicating the matching performance of point features and line features under different conditions. Qualitatively, the model trained using GNN deep weights achieves high matching accuracy. Then compared our method with currently popular point-line feature matching methods on the ETH3D dataset and fully demonstrated the advantages of our improved method in the experimental results shown in S5 Fig.

As illustrated in S5 Fig, the performance of five feature matching methods in terms of algorithmic accuracy is demonstrated under varying recall rate conditions. These conditions include traditional methods (LBD [12], PL [13], SOLD2 [14], L2D2 [15]) and the enhanced method proposed in this paper. The proposed method in this paper demonstrates a marked superiority over competing methods across the entire recall range.

The proposed method demonstrates a clear advantage over current mainstream methods in terms of point-line fusion matching accuracy, as evidenced by the overall trend. The proposed method maintains a high level of accuracy across the entire recall range from 0 to 1, with an initial value close to 1, and remains stable above 0.75 in the high recall region (Recall > 0.8). This enhancement in performance is attributed chiefly to the structure-aware characteristics provided by GNNs and their effective modelling capabilities for the relationships between point features and line structures. Consequently, while this approach effectively detects a greater number of matching pairs, it can also significantly reduce the number of false matches.

In contrast, LBD and PL exhibit a rapid decline in precision within the medium-to-high recall range (0.4–0.9), indicating that they are prone to introducing a large number of false matches when faced with high recall requirements, resulting in instability in precision. SOLD2 demonstrates relatively stable precision in the intermediate range, but its overall values remain lower than those of the method proposed in this paper. L2D2 performs the worst, with its precision dropping below 0.15 when recall reaches 0.5. This study believes this is primarily due to the fact that methods like L2D2 neither utilize keypoint information in the scene nor introduce geometric consistency constraints during the matching process, making it difficult to achieve effective matching performance in this experimental environment.

### 3.3 Public dataset evaluation

Quantitative evaluations were performed on the publicly available high-dynamic TUM dataset in the system, with local feature matching and dynamic exclusion being run.

As illustrated in S6 Fig, the process of point-line feature extraction and matching is demonstrated on a publicly available high-dynamic dataset. The experimental environment is characterized by a substantial presence of rapidly moving objects and structurally complex regions. The results presented in Figure demonstrate the system’s remarkable robustness in point and line feature recognition, accurately extracting structural information from images, particularly in scenarios involving significant geometric changes, prominent edges, or abrupt depth variations. In such contexts, the system exhibits enhanced precision and stability in environmental feature recognition and matching.

Subsequently, a qualitative performance evaluation was conducted on the public dataset. A step-by-step comparison was performed of the original ORB-SLAM3 algorithm (henceforth referred to as ORB), the PL-SLAM algorithm with improved line descriptors and bag-of-words-based cyclic closure description (henceforth referred to as PL), and the algorithm developed by the present authors (henceforth referred to as OURS). The three algorithms were executed on the high-dynamic TUM dataset, and the alignment was performed using the ground truth coordinate axes of the true trajectory. The resulting trajectory plots are displayed in S7 Fig, and the RPY rotation motion sequences are displayed in S8 Fig.

As demonstrated in S7, it is evident that in high-dynamic environments, the pose estimation offset of the original ORB algorithm is the most significant, manifesting considerable trajectory deviation; the pose estimation offset of the PL algorithm is diminished in comparison to ORB, yet there persists moderate trajectory deviation; while the trajectory performance of the OURS algorithm is most analogous to the true trajectory, exhibiting substantial superiority over the preceding two methods. S8 Fig. further validates this conclusion. A comparison of the changes in the rpy angles (roll, pitch, and yaw) reveals that both the ORB and PL algorithms demonstrate significant errors in pose estimation, with the ORB algorithm exhibiting particularly pronounced errors, while the OURS algorithm displays only minor deviations. This finding suggests that the OURS algorithm demonstrates enhanced robustness and accuracy in high-dynamic scenarios, thereby enabling it to more effectively address pose estimation challenges under complex motion conditions.

In the quantitative evaluation experiment, the ATE (absolute trajectory error) and RPE (relative trajectory error) of the aligned true trajectory were selected as the primary evaluation metrics. The maximum error (max), average error (mean), root mean square error (rmse), and standard deviation (std), among others, were then compared. The RPE measurement interval was set to one second, and the tracking time per frame was utilized to measure efficiency in milliseconds.

ATE and RPE are widely adopted metrics for evaluating the trajectory accuracy of visual localization and mapping systems. ATE quantifies the overall deviation of the estimated trajectory from the ground truth in the global coordinate frame, primarily reflecting the accumulated drift over time. The computation generally involves aligning the estimated trajectory to the ground truth to eliminate coordinate discrepancies, followed by calculating the root mean square of the positional deviations across all frames. In contrast, RPE measures the accuracy of local motion estimation by assessing the consistency of relative pose changes between consecutive frames or over a fixed time interval. It captures both translational and rotational deviations between the estimated and true relative motions, thereby indicating how precisely the system tracks short-term movements. RPE is more sensitive to instantaneous drift and motion jitter, revealing the temporal stability of the estimation process. Thus, ATE focuses on the global consistency and long-term accuracy of the trajectory, whereas RPE reflects local motion precision and short-term robustness. The combination of these two metrics provides a comprehensive evaluation of the system’s localization and mapping performance.

As illustrated in S9 Fig. and data in S1 Table, a comparative analysis is presented of the ORB, PL, and OURS algorithms with regard to ATE (m) and RPE (m) metrics.

To illustrate this point, consider the RMSE metric of ATE. It is evident that different algorithms demonstrate marked variations in trajectory estimation accuracy under high-dynamic scenarios. The RMSE of the ORB algorithm is 0.613, that of the PL algorithm is 0.845, while the OURS method achieves 0.416, thus demonstrating a significant advantage in terms of accuracy. A subsequent analysis of the RMSE of the RPE reveals an error of approximately 0.025 for the ORB algorithm, 0.020 for the PL algorithm, and 0.011 for our method, indicating superior accuracy. It is noteworthy that, despite the superior performance of the OURS algorithm in the majority of metrics, its performance in the SSE metric is marginally lower than that of PL-SLAM. Preliminary analysis suggests that this phenomenon may be attributable to the integration of high-precision voxel point cloud maps within the proposed methodology, with the objective of enhancing the geometric constraint information of the scene. While this strategy has been shown to enhance global composition accuracy, it has also been observed to result in a decline in real-time performance when dealing with high-dynamic environments. This is attributed to the substantial computational demands of point cloud processing, which in turn affects the system’s reliability in instantaneous pose estimation. Therefore, the error under discussion is not attributable to a degradation in tracking accuracy itself. Rather, it is more likely to reflect the challenges of balancing real-time performance and accuracy in the algorithm.

Data in S2 and S3 Tables summarize the RMSE performance of various algorithms in terms of ATE and RPE. Among these, Dyna-SLAM [25] and DS-SLAM [26] are the most widely used benchmark algorithms and are commonly employed for performance comparisons in dynamic environments. DIG-SLAM [27] integrates instance segmentation and line segment detection techniques to identify and process dynamic regions. DRV-SLAM [28] detects potential moving targets based on image analysis technology and estimates their instantaneous motion state. Additionally, DG-SLAM [29] and DGS-SLAM [30] introduce Gaussian splash modeling methods to effectively enhance the system’s localization stability in complex scenes. Notably, DN-SLAM [31], a recently proposed scheme, demonstrates strong innovation and practicality in dynamic feature point removal.

In the various typical dynamic datasets, quantitative comparisons of the ATE and RPE of each algorithm were conducted, and qualitative analysis was introduced to reveal the key factors behind the performance differences. As demonstrated in S2 Table, in the “fr3_w_xyz” scenario, our method attains an RMSE of 0.0141, surpassing all comparison methods. A comparison of the RMSE with those of Dyna-SLAM and DIG-SLAM reveals a reduction of approximately 14.5% and 3.4%, respectively, thereby demonstrating the efficacy of the proposed method in accurately estimating attitude in complex environments characterized by translation, rotation, and occlusion interference. In the “fr3_w_static” scenario, the proposed algorithm attained an RMSE of 0.0055, thereby demonstrating superior performance in comparison to all other methods. This is evidenced by a reduction of 31.2% and 9.8%, respectively, in comparison to DS-SLAM and DG-SLAM. This outcome thus substantiates the algorithm’s capacity for geometric consistency mapping, even in static backgrounds. In the “fr3_w_rpy” scenario, the proposed algorithm attained an RMSE of 0.0325, which is lower than the RMSEs of methods such as DIG-SLAM and Dyna-SLAM, with a reduction of approximately 30.5%. The relative error assessment in S3 Table further corroborates this trend. In the “fr3_w_xyz” scenario, OURS attains an RPE of approximately 0.0155, surpassing DIG-SLAM (0.0240) and DG-SLAM (0.0199). This outcome signifies that OURS exhibits reduced relative drift error even in the presence of dynamic object interference. In the “fr3_w_static” scenario, OURS attains an RPE of 0.0089, marginally higher than that of DG-SLAM but still surpassing the majority of methods. This suggests that the system sustains high-precision tracking capabilities even in the absence of dynamic interference. In the “fr3_w_rpy” scenario, OURS achieves an RPE of 0.0355, signifying a reduction of approximately 45%.The results of the study demonstrated that the OURS model outperformed the DIG-SLAM model in terms of rotational motion precision, with an 8% improvement in performance. In the “fr3_w_half” scenario, the OURS model achieved an RPE of 0.0224, representing a 40.7% reduction in error compared to the DGS-SLAM model. This suggests that the fusion strategy employed by the OURS model effectively mitigates the propagation of cumulative errors under semi-dynamic conditions. The efficacy of the proposed algorithm was demonstrated by its attainment of optimal outcomes in both absolute and relative trajectory error metrics on the experimental dataset. The experimental results demonstrate that this method is capable of high trajectory estimation accuracy in dynamic environments, and also exhibits significant robustness in scenes with complex motion interference. In comparison with alternative methodologies, this algorithm has been demonstrated to exhibit consistent localization performance under diverse dynamic configurations, thereby substantiating its extensive adaptability and dependability in practical applications.

### 3.4 Map building performance evaluation

To validate the practical effectiveness of this system in dense mapping, this study selected several different TUM RGB-D datasets for mapping demonstrations, focusing on evaluating the reconstruction accuracy and geometric structure representation capabilities of the voxel map construction method proposed in this paper in actual scenarios. S10 Fig shows a comparison between the dense mapping results of the ORB-SLAM2 algorithm and those of our system in the TUM dataset.

As illustrated in S10 Fig, the results of dense mapping in a non-dynamic environment were obtained using the original ORB-SLAM2 algorithm and the present algorithm, which incorporates a point-line mapping thread. The map generated by ORB-SLAM2 is dense and chaotic, which hinders the ability to discern complete object information. Consequently, these maps are rendered useless. In contrast, the present algorithm generates a dense point cloud map with distinct edges of background objects and unobstructed foregrounds, thereby producing a more precise dense point cloud map. It has been demonstrated that ORB-SLAM2 is incapable of achieving precise point cloud reconstruction of certain intricate objects, including the intricate details of potted plant leaves and the accurate generation of desktop objects. However, the precise dense point cloud reconstructed by the voxel mapping method in this paper is crucial for reconstructing the three-dimensional model of the environment. The point cloud generated by this algorithm is characterized by its high density and clear boundary contours, particularly in structurally significant areas such as desktops, floors and furniture. The system filters and reconstructs depth information for each key frame through a parallel voxel mapping module, effectively removing noise interference while preserving key structural surfaces. It has been demonstrated that all point clouds retain original texture information through color mapping, thereby enhancing the overall map’s interpretability and visual consistency. As demonstrated by experimental comparisons, this algorithm’s dense point cloud construction strategy has been shown to be more effective in the creation of precise, dense point cloud maps within the environment.

### 3.5 Evaluation of overall system robustness

This subsection aims to evaluate the practical usability and robustness of the proposed system from two perspectives: operational efficiency and loop-closing capability. The experiments include: (1) a quantitative assessment of computational complexity and time overhead, and (2) comparative experiments on loop detection effectiveness against several representative baseline algorithms. All quantitative results are reported as mean ± standard deviation, and appropriate statistical significance tests are performed. Each experiment is repeated N=10 times under identical hardware and software environments to obtain reliable statistical measures.

To evaluate the computational performance of the proposed system, a comprehensive runtime analysis was conducted by comparing it with two representative baseline methods, ORB and PL, as well as OURS method. All experiments were conducted under the same hardware and software configurations to guarantee evaluation fairness. The evaluation metrics include: average frame processing time, total runtime for completing the entire sequence, average voxel-to-map integration time, and overall mapping time required for complete reconstruction.

As shown in S11 Fig, from the perspective of time overhead, the proposed OURS method exhibits slightly higher per-frame processing time and total runtime compared with ORB and PL. Taking the f1_floor dataset as an example, the average per-frame processing time of OURS is approximately 0.41 s, which is about 24.2% and 2.5% higher than those of ORB (0.33 s) and PL (0.40 s), respectively. The total runtime of OURS reaches 53.17 s, exceeding ORB (49.87 s) and PL (51.59 s). In the f3_teddy dataset, which contains richer scene details, OURS achieves an average per-frame processing time of 0.038 s, higher than ORB (0.034 s) and PL (0.036 s); its total runtime is approximately 65.30 s, representing increases of about 7.4% and 3.3% over ORB (60.79 s) and PL (63.20 s), respectively. From the perspective of system complexity and mapping quality, the point–line matching time of OURS generally falls “between ORB and PL” in most datasets. For instance, in the low-dynamic f1_floor dataset, the matching time of OURS is 0.014 s, nearly identical to that of PL; while in the f3_teddy dataset with richer geometric features, OURS requires 0.021 s, slightly higher than PL’s 0.018 s. Since the ORB baseline does not include a point–line matching module, only the PL system is referenced for complexity comparison.

In terms of mapping quality, OURS consistently demonstrates a clear advantage across all test datasets. For example, in the f2_kidnap dataset, OURS achieves a mapping quality score of 87.20, which is approximately 6.2% and 11.4% higher than PL (82.10) and ORB (78.30), respectively. In the f3_xyz dataset, OURS attains a mapping score of 57.40, surpassing PL (53.30) and ORB (51.10), indicating its superior accuracy in fine-grained structural reconstruction.

From a system-level perspective, the main reason for the increased time overhead of OURS lies in the voxel mapping module at the front end, which is designed to meet the demands of high-precision reconstruction. This module performs multi-stage computations for each RGB-D frame. First, the depth image is back-projected into a 3D point cloud using the camera intrinsics, followed by bilateral filtering to preserve edge structures; the computational complexity of this step grows linearly with the number of valid pixels. Next, to strengthen geometric constraints in low-texture regions, each 3D point undergoes neighborhood plane fitting, where the covariance matrix is solved via least-squares and the normal vector is extracted—an operation that traverses all valid points and thus incurs considerable computational cost. Finally, the Octree-based voxel update requires real-time maintenance of occupancy states and TSDF values; when loop closure is triggered, the global voxel map must be re-aligned, further increasing the total runtime. In contrast, ORB and PL rely solely on sparse point or line features for pose estimation. Their front ends do not include dense voxel modeling modules, and their computational burden is primarily concentrated in feature extraction and matching, resulting in significantly lower processing times. The additional cost of OURS represents a rational trade-off between high-precision mapping, system complexity, and runtime efficiency. The high computational expense of the voxel-based front end directly translates into improved reconstruction quality: in low-texture scenes, OURS achieves superior reconstruction of tabletop edges and small object contours compared to ORB and PL; in highly textured environments, it also demonstrates stronger detail preservation. Moreover, the global geometric constraints provided by the voxel map help the bag-of-words model filter out “visually similar but geometrically inconsistent” loop candidates, thereby enhancing the overall robustness of the system.

To further complement the system-level computational performance analysis, the loop detection capability of the proposed algorithm was evaluated. Unlike the previous experiment, which mainly focused on runtime efficiency, this part emphasizes the algorithm’s ability to accurately recognize previously visited locations under diverse motion patterns and environmental conditions. By quantitatively analyzing key metrics such as detection precision, recall rate, and the number of missed loop closures, the robustness and reliability of the algorithm in loop detection are comprehensively assessed.

S12 Fig. presents the precision–recall curves for loop detection of the baseline algorithms ORB and PL, as well as the proposed OURS method, on the same high-dynamic dataset sequence. The curves primarily reflect each algorithm’s ability to balance “accurate recognition of previously visited scenes” with “coverage of more repeated scenes,” and the results further demonstrate the superiority of OURS in loop detection robustness.

From the overall trend of the curves, OURS consistently maintains the highest precision across the full recall range, and its precision decreases much less sharply with increasing recall compared to the baseline methods, indicating a better trade-off between accuracy and coverage. Specifically, in the low-recall region (Recall < 0.3), all three algorithms achieve relatively high precision; however, OURS benefits from the global geometric constraints provided by the voxel map, which reduces mismatches at the source, allowing it to achieve high precision even at low recall levels.

In the medium-recall range (0.3 ≤ Recall ≤ 0.7), the performance gap among the algorithms further widens. When Recall reaches 0.5, OURS maintains a stable precision above 0.85, whereas PL drops to 0.65 and ORB falls sharply to 0.45. This discrepancy is primarily attributed to differences in feature representation capabilities: although PL fuses point and line features, it does not model the structural relationships among features, making it susceptible to mismatches in high-dynamic scenarios due to line fragmentation and viewpoint changes; ORB relies solely on point features, which exhibit high repetition in low-texture regions, leading to unstable loop detection. In contrast, OURS employs GNN to achieve structured fusion of point–line features, combined with a cross-graph adhesion strategy that reinforces topological consistency among features. As a result, even as recall increases, most false positives are filtered through structural association, leading to a more gradual decline in precision.

In the high-recall range (Recall > 0.7), the advantage of OURS becomes particularly pronounced. When Recall reaches 0.9, OURS still maintains a precision of 0.72, whereas PL and ORB achieve only 0.62 and 0.51, respectively. This outcome demonstrates the effectiveness of the cross-graph adhesion strategy, which propagates structural information to weak feature nodes through bidirectional cycle matching and cycle consistency loss. Even when some local features lack robustness, reliable matches can still be achieved via global graph structure associations. In contrast, PL and ORB lack such global structural constraints, and at high recall values, numerous false loops are included, causing precision to collapse sharply.

As shown in S13 Fig, regarding loop detection performance across different datasets, the OURS method achieves the best results in both the proportion of missed loop closures and the RMSE of ATE after loop closure, further demonstrating its stability and robustness in complex environments. In terms of missed loop ratios, ORB exhibits an average miss rate of approximately 17.8% across all datasets, PL reduces this to 14.5%, while OURS achieves only 7.2%, corresponding to reductions of 59.6% and 50.3% compared to ORB and PL, respectively. This significant improvement is primarily attributed to the synergistic effect of voxel mapping and the GNN-based structured fusion strategy. Voxel modeling provides geometric consistency constraints at the global scale, mitigating loop misses caused by feature drift or abrupt viewpoint changes, while the GNN performs multi-layer perception and information aggregation over the topological structure of heterogeneous point–line features, enabling robust feature re-identification even in degraded scenarios such as low-texture regions or motion blur.

In terms of RMSE of ATE for loop detection, the average errors of ORB and PL are approximately 0.091 m and 0.076 m, respectively, whereas OURS consistently achieves around 0.048 m, representing an overall improvement of approximately 36%–47%. Notably, in sequences with significant viewpoint changes and occlusions, such as f2_kidnap, the RMSE of ORB can exceed 0.12 m, reflecting substantial false loop closures and global optimization drift. In contrast, OURS effectively mitigates registration drift of weak feature nodes through the cross-graph adhesion strategy, resulting in significantly reduced pose estimation errors after loop closure. It is worth noting that in geometrically complex datasets such as f3_teddy, although OURS exhibits slightly higher local detection latency, it still maintains the lowest ATE errors and the fewest missed loops, demonstrating the system’s generalization capability in high-texture-redundant environments and its robustness to noise. These results indicate that the proposed algorithm not only achieves superior precision and recall in loop detection, but also significantly reduces trajectory drift caused by missed or mismatched loops at the system level, ensuring consistency in global optimization and reproducibility of the mapping results.

## 4. Conclusions

Our paper proposes a visual SLAM system that integrates voxel mapping and GNN based point-line feature fusion, aimed at addressing the issue of insufficient robustness in traditional feature matching under low-texture, dynamic interference, and unstructured scenes. The system is based on the ORB-SLAM3 framework, incorporating a custom voxel map parallel construction mechanism in the frontend to enhance spatial modeling capabilities in low-texture regions. In the backend, GNN is employed to achieve structured joint modeling and feature matching between point and line features, effectively improving registration accuracy and matching robustness. A cross-graph linkage strategy further reinforces semantic consistency across views, maintaining stable performance even under complex conditions such as occlusion and viewpoint changes. Experimental results show that our system significantly outperforms existing point-line fusion methods in terms of accuracy, robustness, and mapping quality across multiple public datasets, particularly in high-dynamic scenes, validating the effectiveness and adaptability of the proposed approach.

## 5. Summary and outlook

The proposed system is a visual SLAM system that integrates voxel mapping with GNN point-line features. The aim of this integration is to improve feature matching accuracy and system robustness in low-texture, dynamic environments. The experimental results demonstrate the efficacy of the proposed method in reducing absolute and relative trajectory errors when confronted with challenges such as dynamic objects, occlusions, and lighting changes. This investigation signifies the significant advantages of the proposed method over existing algorithms. The voxel mapping module enhances the geometric consistency of the map, while the GNN improves the matching efficiency and accuracy of point-line features. In complex dynamic scenes, the system demonstrates strong robustness and high-precision localization capabilities.

Future research can be further expanded in several directions. Firstly, further integration of surface features and point-line features has the potential to enhance the system’s structural modelling capabilities in more complex environments, particularly in indoor-outdoor mixed scenarios. Secondly, the integration of multi-modal sensors, such as LiDAR and IMU, has the potential to further enhance the system’s robustness under extreme lighting conditions, particularly in scenes characterized by an absence of texture or a paucity of texture. It is evident that the GNN module, which is based on deep learning, continues to exhibit a high degree of computational overhead. It is recommended that future research efforts be directed towards the exploration of lightweight models and adaptive training strategies. The objective of this research is to achieve more efficient real-time deployment, thereby addressing the requirements of mobile robots and autonomous systems.

## Supporting information

S1 Fig (a) Original RGB point cloud map and (b) planar feature map under the front-end voxel mapping module. For simplicity, the voxel map is constructed using an octree model with a resolution of 1 cm.
(TIF)

S2 Fig Summary of the GNN point-line fusion strategy. key points and line features are extracted from the two images, along with corresponding dense descriptors, and unified into two-line frames. (TIF)

S3 Fig Experimental setup diagram. (TIF)

S4 Fig Experimental process of the back-end depth map neural network fusion point-line matching strategy. Based on GNN-trained weights for deep point-line feature detection and matching for different experimental images.
(TIF)

S5 Fig Algorithm accuracy of five feature matching methods under different recall conditions. (TIF)

S6 Fig Point-line feature extraction and matching process performed on a publicly available high-dynamic dataset. (TIF)

S7 Fig Algorithm comparison trajectory diagram, with dashed lines representing actual trajectories. Blue lines representing the original ORB algorithm trajectories, green lines representing PL + , and red lines representing the trajectories of our algorithm is closer to the actual trajectories.
(TIF)

S8 Fig Representation of the motion sequence of the heading angle in the RPY coordinate system, with the plots from the top to the bottom representing the tracking error curves of roll, pitch and yaw angles over time. (TIF)

S9 Fig Comparison of ATE and RPE trajectories of three algorithms under a high-dynamic public dataset. (TIF)

S10 Fig Comparison of mapping results. Comparison of high-precision point cloud reconstruction results between ORB-SLAM2 (a) and this algorithm (b) on the fr2_desk dataset.
(TIF)

S11 Fig Comparison of overall system robustness across different datasets. (TIF)

S12 Fig Precision–Recall curves of different algorithms on the same high-dynamic dataset sequence. (TIF)

S13 Fig Comparison of missed loop closures and loop detection ATE RMSE across different datasets. (TIF)

S1 Table (DOCX)

S2 Table (DOCX)

S3 Table (DOCX)
