# Supplementary material for: Integrating voxel mapping with deep network-based point-line feature fusion for robust SLAM
Source: PLoS One. 2026 Jan 2;21(1):e0337917. doi: 10.1371/journal.pone.0337917 (PMC12758739; doi:10.1371/journal.pone.0337917)
Supplement: S1 Table — (DOCX) [file pone.0337917.s014.docx]

**S14 Table**

| index | ORB-SLAM3 | | PL-SLAM | | OURS | |
| --- | --- | --- | --- | --- | --- | --- |
|  | ATE(m) | RPE(m) | ATE(m) | RPE(m) | ATE(m) | RPE(m) |
| max | 1.253 | 0.117 | 1.148 | 0.078 | 0.046 | 0.043 |
| mean | 0.547 | 0.031 | 0.328 | 0.016 | 0.013 | 0.009 |
| median | 0.451 | 0.019 | 0.247 | 0.012 | 0.011 | 0.007 |
| min | 0.056 | 0.006 | 0.014 | 0.008 | 0.002 | 0.001 |
| rmse | 0.613 | 0.025 | 0.845 | 0.020 | 0.416 | 0.011 |
| sse | 298.640 | 0.549 | 54.381 | 0.273 | 115.350 | 0.121 |
| std | 0.286 | 0.014 | 0.256 | 0.012 | 0.008 | 0.006 |
