# Supplementary material for: Integrating voxel mapping with deep network-based point-line feature fusion for robust SLAM
Source: PLoS One. 2026 Jan 2;21(1):e0337917. doi: 10.1371/journal.pone.0337917 (PMC12758739; doi:10.1371/journal.pone.0337917)
Supplement: S2 Table — (DOCX) [file pone.0337917.s015.docx]

**S15 Table**

| Datasets series | Dyna‐SLAM(m) | DS‐SLAM(m) | DG‐SLAM(m) | DGS‐SLAM(m) | DIG‐SLAM(m) | OURS(m) |
| --- | --- | --- | --- | --- | --- | --- |
| fr3_w_xyz | 0.0165 | 0.0248 | 0.0165 | 0.0413 | 0.0146 | 0.0141 |
| fr3_w_static | 0.0064 | 0.0080 | 0.0061 | 0.0062 | 0.0068 | 0.0055 |
| fr3_w_rpy | 0.0359 | 0.4618 | 0.0432 | - | 0.0468 | 0.0325 |
| fr3_w_half | 0.0254 | 0.0310 | - | 0.0550 | 0.0240 | 0.0239 |
