# Supplementary material for: Integrating voxel mapping with deep network-based point-line feature fusion for robust SLAM
Source: PLoS One. 2026 Jan 2;21(1):e0337917. doi: 10.1371/journal.pone.0337917 (PMC12758739; doi:10.1371/journal.pone.0337917)
Supplement: S3 Table — (DOCX) [file pone.0337917.s016.docx]

**S16 Table**

| Datasets series | Dyna‐SLAM(m) | DS‐SLAM(m) | DG‐SLAM(m) | DGS‐SLAM(m) | DIG‐SLAM(m) | OURS(m) |
| --- | --- | --- | --- | --- | --- | --- |
| fr3_w_xyz | 0.0240 | 0.0335 | 0.0199 | 0.0230 | 0.0240 | 0.0155 |
| fr3_w_static | 0.0112 | 0.0108 | 0.0087 | 0.0102 | 0.0114 | 0.0089 |
| fr3_w_rpy | 0.0415 | 0.1604 | 0.1421 | 0.0421 | 0.0655 | 0.0355 |
| fr3_w_half | 0.0401 | 0.0301 | 0.0255 | 0.0378 | 0.0355 | 0.0224 |
